# The Value of HALP Score, SII, and SIRI in Predicting the Need for Intensive Care and Assessing Disease Severity in Pediatric Diabetic Ketoacidosis Cases

**DOI:** 10.3390/children12111445

**Published:** 2025-10-24

**Authors:** Muhammed Selçuk Sinanoğlu, Turgut Dolanbay, Bilgehan Demir, Süleyman Nogay, Can Celiloğlu, Muhammed Eyyüb Polat

**Affiliations:** 1 Department of Pediatrics, Faculty of Medicine, Malatya Turgut Özal University, 44210 Malatya, Türkiye; 2 Department of Emergency Medicine, Faculty of Medicine, Malatya Turgut Özal University, 44210 Malatya, Türkiye; turgut.dolanbay@ozal.edu.tr (T.D.); bilgehan.demir@ozal.edu.tr (B.D.); suleyman.nogay@ozal.edu.tr (S.N.); eyyubpolat97@gmail.com (M.E.P.); 3 Pediatric Endocrinology Specialist, Malatya Training and Research Hospital, 44000 Malatya, Türkiye; cceliloglu@cu.edu.tr

**Keywords:** critical care, diabetic ketoacidosis, HALP score, inflammatory index, pediatrics

## Abstract

**Objective**: The aim of this study was to evaluate whether Hemoglobin, Albumin, Lymphocyte, and Platelet (HALP) scores, the Systemic Immune-Inflammation Index (SII), and the Systemic Inflammatory Response Index (SIRI) can predict intensive care unit (ICU) or inpatient admissions in pediatric diabetic ketoacidosis (DKA) cases and to determine their sensitivity and specificity. **Methods**: This retrospective study included 39 pediatric patients (<18 years) diagnosed with DKA (pH < 7.3, HCO_3_ < 15). HALP, SII, SIRI, and urine ketone values were collected from medical records. Statistical analyses included ROC curve analysis, correlation tests, and appropriate parametric or non-parametric comparisons to evaluate associations with 30-day outcomes. **Results**: The median age was 13 years (IQR: 8–15), 56.4% were male, and 64.1% required ICU monitoring. ICU patients had significantly lower pH (*p* = 0.005) and HCO_3_ (*p* = 0.012) and significantly higher monocyte, SII, and SIRI values (all *p* ≤ 0.018). ROC analysis showed SIRI had the highest predictive power for ICU admission (cut-off: 3911; sensitivity: 76%; specificity: 85.7%), followed by SII. HALP scores did not demonstrate any value in assessingdisease severity for predicting ICU admission (AUC = 0.25). **Conclusion**: SIRI and SII are reliable predictors of ICU admission in pediatric DKA. HALP scores do not predict ICU admission and should not be considered a marker of disease severity. Incorporating SIRI and SII into clinical decision-making may improve early risk stratification. Prospective multicenter studies are warranted to validate these results.

## 1. Introduction

Type 1 diabetes mellitus (T1DM) is a chronic autoimmune disease characterized by insulin deficiency due to pancreatic β-cell destruction. It commonly affects children and adolescents and is associated with various acute and chronic complications. The global prevalence of T1DM continues to increase, and diabetic ketoacidosis (DKA) represents one of its most frequent and severe acute complications [1]. T1DM incidence is increasing globally by approximately 3–5% each year, particularly in younger age groups, and it poses a significant public health concern due to its lifelong impact and risk of acute metabolic decompensation [2]. The autoimmune nature of the disease leads to the destruction of β-cells, resulting in an absolute dependence on insulin therapy and vulnerability to metabolic crises such as DKA [3]. Every year, 4.6–13.4 of every 1000 people with diabetes, varying based on age and sex, present to hospitals with a clinical picture of DKA [4], while mortality and morbidity rates increase at younger ages [4,5]. Despite significant advances in diabetes management and insulin therapy, DKA remains a major cause of hospitalization and mortality among children with T1DM [6]. Early recognition of patients at risk of severe DKA or intensive care unit (ICU) admission is therefore crucial for reducing complications and improving outcomes [6]. Inflammation, immobilization, muscle loss, and elevated cytokines also pave the way for DKA formation by disrupting the peripheral glucose consumption of patients [7].

The Hemoglobin, Albumin, Lymphocyte, and Platelet (HALP) score is a unique nutritional–immune marker that combines immune function parameters including lymphocyte and platelet counts with nutritional state parameters including hemoglobin and albumin levels [8,9]. Moreover, the Systemic Immune–Inflammation Index (SII) and the Systemic Inflammatory Response Index (SIRI) are important inflammatory indices that represent inflammatory response and are used in the assessment of the follow-up and disease severity of several diseases [10,11]. These indices are advantageous because they can be easily calculated from routine blood tests, allowing clinicians to rapidly assess inflammatory status without additional cost or time. Their potential value in metabolic emergencies such as DKA has recently gained interest, as systemic inflammation plays a critical role in the pathophysiology and severity of the disease [12]. While these indices have been extensively studied in oncology, cardiovascular disease, and critical illness, demonstrating value for evaluating disease severity and outcomes such as mortality, treatment response, and postoperative recovery [8,9,10,11], their application in type 1 diabetes—particularly in pediatric diabetic ketoacidosis—remains limited. Only a few studies have explored the relevance of SII or SIRI in pediatric metabolic or endocrine emergencies, and no large-scale investigations have assessed HALP in this patient population. Known limitations of these indices include susceptibility to acute changes in hematologic parameters caused by infection, dehydration, or other concurrent conditions, as well as potential variability arising from pediatric age-related physiological differences. Despite these constraints, their low cost, accessibility, and integration of multiple physiological dimensions make them promising candidates for early risk stratification tools in pediatric DKA [9,10,11].

Given the scarcity of evidence in this field, investigating the clinical value of HALP, SII, and SIRI in assessing disease severity in pediatric DKA could contribute valuable insight into early clinical decision-making and help identify children requiring intensive monitoring or intervention. The primary purpose of our study was to determine whether blood parameters could be used to predict intensive care or inpatient clinic admissions in pediatric DKA patients. The secondary purpose of the study was to identify the sensitivity and specificity of HALP scores, SII values, and SIRI values based on the intensive care admission statuses of patients and examine whether these scores and indices could be used to determine the intensive care indications of pediatric patients diagnosed with DKA in emergency services and as criteria that can be interpreted by clinicians.

## 2. Material and Methods

### 2.1. Ethical Approval and Study Design

This retrospective, single-center, observational study was conducted after obtaining approval from the Non-Invasive Clinical Research Ethics Committee of Malatya Turgut Özal University, Faculty of Medicine (Approval No: 270536243; Date: 26 February 2025). The study was designed and reported in accordance with the STROBE (Strengthening the Reporting of Observational Studies in Epidemiology) guidelines, and the completed STROBE checklist has been included as Supplementary Material. Between 15 January 2020 and 15 January 2025, pediatric patients who presented to the Emergency Department of Malatya Research and Training Hospital and had a prior diagnosis of Type 1 diabetes mellitus (T1DM) were retrospectively evaluated. Patients with pH < 7.3 and HCO_3_ < 15 mmol/L were classified as having diabetic ketoacidosis (DKA), and these individuals constituted the study population.

### 2.2. Study Sample

The inclusion criteria were (1) diagnosis of Type 1 diabetes mellitus, (2) age under 18 years, (3) diagnosis of diabetic ketoacidosis (DKA), and (4) hospital admission for treatment. The exclusion criteria were missing or incomplete hospital records, ongoing anticoagulant or coagulant therapy, referral to an external hospital after emergency department evaluation, and voluntary discharge against medical advice. In total, the data of 44 pediatric patients were accessed; however, 39 patients met all inclusion criteria and were included in the final analysis.

### 2.3. Dependent and Independent Variables

The dependent variable of the study was intensive care unit (ICU) admission status. The independent variables included hematological, biochemical, and inflammatory indices, such as the following: hemogram parameters: leukocytes (WBC), hemoglobin (Hgb), hematocrit (Hct), platelets (PLT), mean corpuscular volume (MCV), and neutrophil percentage (%Neu); biochemical parameters: C-reactive protein (CRP), urea, creatinine (Cr), aspartate aminotransferase (AST), alanine aminotransferase (ALT), albumin (Alb), glucose (Glc), sodium (Na), potassium (K), chlorine (Cl), and calcium (Ca); inflammatory indices: Systemic Immune-Inflammation Index (SII), Systemic Inflammatory Response Index (SIRI), and Hemoglobin-Albumin-Lymphocyte-Platelet (HALP) score. Urine ketone levels were recorded semi-quantitatively as “+3” using a urine dipstick test. All biochemical analyses were performed using the Beckman Coulter AU5800 analyzer (Beckman Coulter Inc., Indianapolis, IN, USA), and complete blood counts were obtained using the Sysmex XN-1000 hematology analyzer (Sysmex Corporation, Kobe, Japan).

### 2.4. Calculation of Inflammatory Indices

All indices were calculated according to previously validated formulas in the literature: HALP score = (Hemoglobin [g/L] × Albumin [g/L] × Lymphocyte count [10^9^/L])/Platelet count [10^9^/L]; SII (Systemic Immune-Inflammation Index) = (Neutrophil count × Platelet count)/Lymphocyte count; SIRI (Systemic Inflammatory Response Index) = (Neutrophil count × Monocyte count)/Lymphocyte count. All blood samples were drawn upon emergency department admission, and analyses were performed using standardized automated laboratory systems to minimize pre-analytical variability.

### 2.5. Statistical Analysis

Statistical analyses were performed using IBM SPSS Statistics version 27 (IBM Corp., Armonk, NY, USA). The Shapiro–Wilk test was used to evaluate the normality of continuous data. Descriptive statistics are presented as mean ± standard deviation (SD) or median with interquartile range (IQR; 25th–75th percentiles) for continuous variables, and as frequency and percentage for categorical variables. Comparisons between ICU and non-ICU groups were made using the independent samples *t*-test for normally distributed variables and the Mann–Whitney U test for non-normally distributed variables. Correlations between quantitative variables (blood parameters and inflammatory indices) and clinical outcomes (e.g., ICU stay duration) were assessed using Pearson or Spearman correlation analysis, as appropriate. Receiver Operating Characteristic (ROC) curve analysis was performed to evaluate the predictive performance of HALP, SII, and SIRI scores for ICU admission. The Youden Index was applied to determine optimal cutoff values, and sensitivity and specificity were reported accordingly. A *p*-value < 0.05 was considered statistically significant.

## 3. Results

The median age of the patients was 13 (years) (IQR: 8–15), and 56.4% of them were male. It was determined that 64.1% of the patients were monitored in intensive care, and these patients were classified as moderate and severe DKA. None of our patients died. Table 1 presents the SII and SIRI values, HALP scores, and blood parameter values [parametric: mean ± standard deviation (SD); non-parametric: median and IQR] of the patients.

There was no significant relationship between the intensive care hospitalization statuses of the patients and their Hgb (g/dL), Hct (%), lymphocyte (/μL), PLT (103/μL) and pCO_2_ (mm Hg) values (respectively, *p*: 0.633, *p*: 0.316, *p*: 0.826, *p*: 0.406, *p*: 0.385, and *p*: 0.984). The HCO_3_ values of the patients who were admitted to intensive care were significantly lower than the values of those who were not admitted (*p*: 0.012). No significant relationship was found between intensive care hospitalization status and urea (mg/dL), creatinine (mg/dL), albumin (g/dL), Na (mmol/L), Ca (mg/dL), Cl (mmol/L), or K (mEq/L) values (respectively, *p*: 0.917, *p*: 0.586, *p*: 0.821, *p*: 0.453, *p*: 0.354, *p*: 0.664, and *p*: 0.888). The patients who were admitted to intensive care units had lower HALP scores, while this difference was not statistically significant (*p*: 0.06). There was no significant relationship between intensive care admission status and WBC (103/μL), MCV (fL), %neu, or neutrophil count (/μL) values (respectively, *p*: 0.272, *p*: 0.682, *p*: 0.736, and *p*: 0.046). Monocyte counts (/μL) were significantly higher in the group of patients who were admitted to intensive care units (*p*: 0.018). The group of patients who were admitted also had significantly lower pH values (*p*: 0.005). Intensive care admission status was not significantly associated with the lactate (mmol/L), fraction of carboxyhemoglobin (FCOHb), glucose (mg/dL), AST (U/L), and ALT (U/L) values of the patients (respectively, *p*: 0.272, *p*: 0.419, *p*: 0.598, *p*: 0.11, and *p*: 0.208). The group of patients who were admitted to intensive care units had significantly higher SIRI and SII values (respectively, *p*: 0.001 and *p*: 0.001). Intensive care admission status was not significantly associated with the urine ketone or urine glucose levels of the patients (respectively, *p*: 0.578 and *p*: 0.203). The relationships between the intensive care hospitalization statuses of the patients and their blood parameters, inflammatory index values, and HALP scores are given in Table 2.

The intensive care hospitalization durations of the patients had moderate positive correlations with their SII values (r: 0.451 *p*: 0.004), SIRI values (r: 0.513 *p*: 0.001), monocyte counts (r: 0.469 *p*: 0.003), and neutrophil counts (r: 0.323 *p*: 0.045) and moderate negative correlations with their pH (r: −0.463, *p*: 0.003) and HCO_3_ (r: −0.4 *p*: 0.012) values. HALP scores, lymphocyte counts, platelets, albumin, WBC, and FCOHb were not significantly correlated with hospitalization durations. The results of the correlation analyses are shown in Table 3.

In decisions to admit pediatric DKA patients to intensive care units, with a cut-off point of 5.2, HALP scores had 28% sensitivity and 72% specificity in a 95% confidence interval. Furthermore, in these decisions, with a cut-off point of 2272, SII values had 72% sensitivity and 85.7% specificity in a 95% confidence interval. In the same context, with a cut-off point of 3911, SIRI values had 76% sensitivity and 85.7% specificity in a 95% confidence interval. Accordingly, SIRI was the index with the highest sensitivity and specificity values in the prediction of intensive care admission decisions.

The sensitivity and specificity values of the examined inflammatory indices and HALP scores in terms of the intensive care admissions of the patients are given in Table 4.

**Figure 1:**
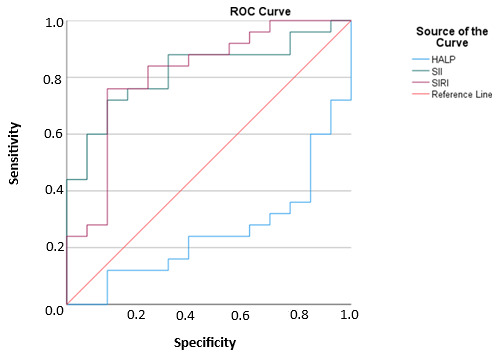
Graphical Representation of the ROC Curve for Inflammatory Indices and HALP Score Based on ICU Admission Status in DKA Patients.

The graphical representation of the ROC curve of the examined inflammatory indices and HALP scores in terms of the intensive care admissions of the patients is given in Figure 1.

The regression analysis demonstrated a statistically significant negative correlation between inflammatory indices (SII, SIRI) and both pH and HCO_3_ levels. Specifically, the association between pH and SII (β = −0.000023, R^2^ = 0.1196, *p* = 0.031) was modest but significant, indicating that elevated systemic immune-inflammation index (SII) values were associated with lower pH levels. A stronger association was observed between pH and SIRI (β = −0.010986, R^2^ = 0.2926, *p* = 0.0004), suggesting that systemic inflammatory response index (SIRI) had a greater explanatory power for pH variability compared to SII.

In terms of metabolic parameters, the relationship between HCO_3_ and SII (β = −0.000191, R^2^= 0.159, *p* = 0.0437) reached statistical significance, although the effect size remained limited. Conversely, the regression between HCO_3_ and SIRI (β = −0.198028, R^2^ = 0.1900, *p* = 0.0055) demonstrated a stronger and more robust inverse association, further supporting the clinical relevance of SIRI in reflecting metabolic acidosis severity.

**Figure 2:**
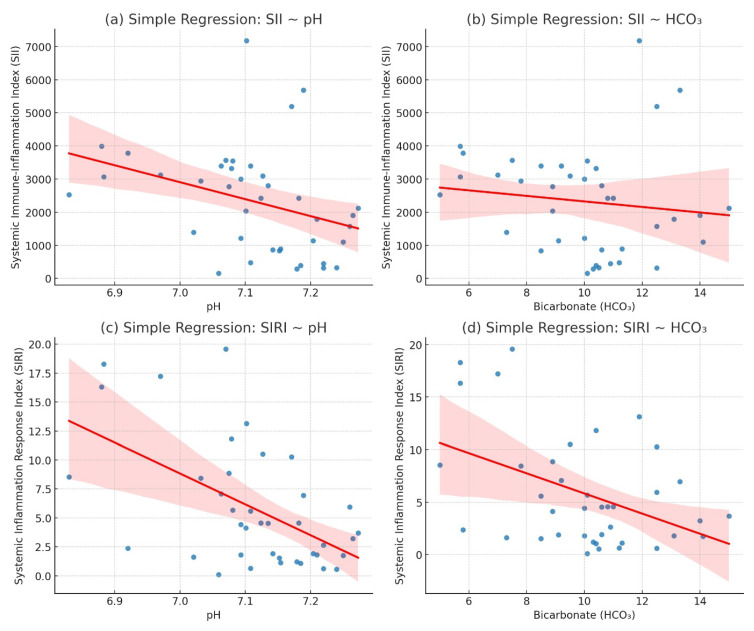
Graphical Representation of the Regression Analysis Between Inflammatory Indices and pH and HCO_3_.

Overall, these findings indicate that SIRI exhibits a more consistent and clinically relevant correlation with both pH and HCO_3_ levels compared to SII, highlighting its potential as a reliable inflammatory biomarker in the assessment of acid–base disturbances. The negative regression coefficients across all models suggest that higher inflammatory activity, quantified by these indices, is associated with worsening acidosis (Table 5, Figure 2).

The coefficient for HCO_3_ was negative (B = −0.423, OR = 0.65), indicating a trend suggesting that higher bicarbonate levels were associated with a lower likelihood of ICU admission; however, this relationship was not statistically significant (*p* = 0.272). A positive trend was observed for SII (B = 0.00093, OR = 1.0009), indicating that an increase in SII values was associated with a minimally higher risk of ICU admission; nevertheless, this finding did not reach statistical significance (*p* = 0.114). No significant relationship was found between the SIRI variable and ICU admission (*p* = 0.885). The pH value had a negative coefficient (B = −0.689, OR = 0.50), suggesting that lower pH might be associated with a higher probability of ICU admission; however, this result was also not statistically significant (*p* = 0.942). All data were analyzed at a 95% confidence level, and the results of the multivariate logistic regression analysis are presented in Table 6.

## 4. Discussion

In recent years, the analysis of medical record data using various information technologies has gained importance in supporting clinical decision-making processes. In this study, we aimed to investigate the role of systemic inflammation indices in predicting intensive care hospitalization needs in pediatric DKA patients. Our results revealed that these indices offered significant predictive intervals and could be used in deciding whether to admit these patients to intensive care units. HALP scores were lower in the patients who were admitted to intensive care units, while this difference was not statistically significant. This study is one of the few investigations to explore the prognostic value of HALP, SII, and SIRI in pediatric DKA cases. It expands the understanding of inflammatory indices beyond adult metabolic diseases by demonstrating their potential relevance in pediatric endocrinologic emergencies.

The mortality rates of children with DKA vary from 0.15% to 0.35% in developed countries such as Canada, the United States, and the United Kingdom, while these rates are in the range of 3.4–13.4% in developing countries such as India, Pakistan, and Bangladesh [13,14]. In a prospective study of 114 pediatric DKA patients, Baloch et al. reported that mortality was not a rare outcome in DKA patients. The mortality rates of DKA patients are higher in developing countries [14]. Previous studies in pediatric DKA have predominantly focused on biochemical and clinical predictors such as pH, HCO_3_, serum lactate, and severity scores (e.g., PRISM III, Pediatric Risk of Mortality [PRISM] score) rather than composite inflammatory indices. For example, Nikhila et al. demonstrated in a tertiary care cohort that PRISM III scores were strongly associated with the need for intensive care admission and adverse outcomes in pediatric DKA patients, underscoring the prognostic value of comprehensive clinical scoring systems [15]. Similarly, Baloch et al., in a prospective study of 114 pediatric DKA patients, reported that mortality and length of ICU stay were significantly predicted by severity scores incorporating neurological status, acid-base balance, and comorbidity burden [14]. Cerebral edema, acute kidney failure, sepsis, and shock are the most frequently encountered causes of mortality in DKA patients, and these complications are more prevalent in developing countries [14,15]. The higher rates of DKA-related mortalities in developing countries in comparison to developed countries may be explained by factors such as delayed diagnosis, limited access to healthcare services, and inadequate treatment infrastructures (e.g., limited intensive care bed capacity). This situation indicates that DKA is an acute metabolic emergency that requires early diagnosis and effective management, and the early identification of cases that require intensive care can contribute to the treatment process.

The practicality of systemic inflammation indices in the prediction of prognosis in various diseases has been studied before. In a study of 401 patients being monitored in intensive care with the diagnosis of sepsis, Xu et al. showed that high SIRI values were associated with poor prognosis, severe sepsis, and increased mortality [16]. Jia et al., who examined the data of 10,764 acute kidney failure (AKF) patients, reported that the group of patients with high SII values had longer intensive care monitoring and hospitalization times, they were diagnosed with AKF at further stages of the course of AKF, and they were in more need of renal replacement treatments [17]. On the other hand, there is limited information about the usage of these indices in pediatric DKA patients, and our study is one of the very few reports on this topic. According to our results, SIRI and SII values were significantly higher in the group of patients who needed intensive care hospitalization (*p* = 0.001). In the ROC curve analysis, the cut-off point of SII was found to be 2272, with 72% sensitivity and 85.7% specificity, whereas the cut-off point of SIRI was found to be 3911, with 76% sensitivity and 85.7% specificity. However, given the relatively small cohort size, these cut-off values should be interpreted with caution, as they are preliminary findings that require confirmation in larger, multi-center prospective studies. In the present study, elevated SII and SIRI values were significantly associated with ICU admission, supporting their clinical relevance as inflammatory biomarkers in metabolic crises such as DKA.

These results suggest that both indices may be clinically useful in determining the need for intensive care in patients with DK. Kocaoğlu et al. reported that HALP scores were not sufficient to predict short- and long-term prognosis in 101 patients with acute heart failure [18]. In a study including 52 patients with adenosquamous carcinoma (ASC), Zhang et al. demonstrated that preoperative HALP scores could potentially serve as a useful biomarker for evaluating postoperative outcomes in ASC patients [19]. In the literature, low HALP scores have generally been associated with malnutrition, immune suppression, and increased inflammatory burden, and are therefore considered valuable biomarkers for predicting mortality and morbidity [18,19]. In our study, HALP scores in the intensive care unit (ICU) group were lower compared to those in the non-ICU group; however, this difference did not reach statistical significance (*p* = 0.06). Receiver operating characteristic (ROC) curve analysis yielded a cut-off value of 5.2 for HALP scores, with a sensitivity of 28% and specificity of 72%. The reported area under the curve (AUC) for the HALP score was 0.25, indicating a negative predictive value. This unexpectedly low AUC value may be related to sample size limitations and variability within the study population and therefore should be interpreted with caution rather than as a meaningful biological finding. These findings indicate that, although HALP is recognized as a valuable marker for nutritional and inflammatory status in adults, its prognostic applicability in pediatric metabolic disorders such as DKA appears limited. This may be due to developmental differences in immune and metabolic responses between children and adults.

In a study of 100 patients brought to the hospital with the diagnosis of DKA, Işık et al. reported that as pH and HCO_3_ values increased, clinical recovery times became shorter, and the prognosis became more favorable [20]. Khan et al., who performed a study with 922 patients diagnosed with DKA, found lower pH and HCO_3_ values in all patients who were admitted to intensive care units in comparison to those who were not admitted [21]. In our study, the patients who required intensive care admission had significantly lower pH (*p* = 0.005) and HCO_3_ (*p* = 0.012) values. These results reinforce the established association between metabolic acidosis severity and intensive care requirement in DKA and emphasize that inflammation-based indices may provide additional prognostic insight when interpreted alongside acid-base parameters.

In our cohort, the median WBC count was elevated (17.1 × 10^3^/μL; IQR: 9.8–22.7). While this leukocytosis may partly reflect the physiological stress response and dehydration associated with diabetic ketoacidosis, it may also be attributable to concurrent infections. Several studies, including one previously conducted by our group, have reported an increased incidence of infection in patients presenting with DKA [20,21]. Infections—particularly respiratory and urinary tract infections—are recognized as both precipitating factors and complications of DKA, and systemic inflammation may exacerbate metabolic decompensation. However, in our dataset, systematic microbiological or clinical confirmation of infection was not available for all patients, precluding a reliable subgroup analysis. Consequently, we cannot definitively determine the proportion of cases in which leukocytosis was infection-related versus DKA-related. Future prospective studies with standardized infection screening protocols are warranted to clarify the role of concurrent infections in influencing inflammatory markers and prognosis in pediatric DKA.

In their study on 2684 patients who were admitted to intensive care units, Liu et al. found a strong relationship between elevated ALT levels and mortality and asserted that ALT values should be closely monitored in these patients [22]. In another study, which was performed by Stadler et al. with 9226 patients diagnosed with T1DM, it was demonstrated that the group of patients with high AST and ALT values had a higher cardiovascular risk profile and poorer glycemic control [23]. Although there is usually a positive relationship between elevated ALT levels and mortality in the general population and T2DM patients, there are also studies showing no significant relationship between these two variables [22,24]. AST and ALT are specific markers of hepatocyte damage. While the irregular hepatic lipid metabolism of diabetic patients may lead to liver damage, the risk of cirrhosis in patients with long durations of DM diagnosis is higher. DM also contributes to a poorer prognosis in liver diseases [22]. In this study, we did not find a significant relationship between AST and ALT values and intensive care hospitalizations. The relationship between DM and liver conditions may be considered a two-way vicious cycle. The oxidative stress caused by insulin resistance and hyperglycemia can damage liver tissue, while liver dysfunctions may also make glycemic control more difficult. The fact that the physiopathological mechanisms and the compensatory responses given to metabolic disorders in the pediatric age group are different compared to those in adults may lead the liver to underreact in cases of pathological reactions such as oxidative stress in this age group [25,26]. It is also known that chronic diseases like cardiovascular comorbidities are less commonly observed in children compared to adults. Lower rates of comorbidities may also reduce the severity of liver damage in this age group. Our results suggested that age-related differences should be considered in the interpretation of biochemical markers in the management of DKA [27].

There are several limitations to our study. First, this was a single-center retrospective study with a relatively small sample size, which may limit the statistical power and generalizability of the findings. Second, as with all retrospective studies, potential sources of bias such as missing data, differences in clinical management protocols, and inconsistent documentation may have influenced the results. Although all patients were managed according to institutional DKA treatment protocols, important clinical variables—such as the Glasgow Coma Scale (GCS) score, presence of comorbidities, and underlying causes of insulin deficiency—were not consistently available in the hospital database. Consequently, these factors could not be incorporated into the multivariate analyses, and their absence may have partially affected the interpretation of outcomes. Finally, because the study was conducted in a single tertiary center, the findings should be validated in larger, multicenter prospective studies to confirm their external validity and clinical applicability.

## 5. Conclusions

In pediatric DKA patients, SIRI and SII are reliable parameters in the prediction of intensive care admission needs. HALP scores, on the other hand, have a lower predictive value in the pediatric population. The integration of these indices into decision-making processes in intensive care and emergency settings can make significant contributions to patient management. Our findings should be supported by prospective studies with larger samples.

## Figures and Tables

**Table 1:** Parametric (mean ± SD) and Non-parametric (median and IQR) Blood Parameters, SII and SIRI, HALP Score in DKA Patients.

	Mean ± SD		Median	IQR (25–75%)
Hb (g/dL)	14.2 ± 1.4	Wbc (10^3^/uL)	17.1	9.8–22.7
Hct (%)	42.6 ± 3.6	Mcv (fL)	81	77.8–85.7
Lymphocyte (mcL)	3 ± 1.1	Neu %	72.7	61.3–82.6
Plt (10^3^/uL)	423.7 ± 115.2	Neutrophil (mcL)	15.3	7.1–20.4
		Monocyte (mcL)	0.8	0.6–1.1
PCO_2_ (mm Hg)	26.1 ± 7.4	pH	7.12	7.07–7.18
HCO_3_ (mmol/L)	10 ± 2.4	Lactate (mmol/L)	2.6	1.7–3.7
Urea (mg/dL)	30.2 ± 12	FCoHb	0.8	0.4–1.2
Creatinine (mg/dL)	0.8 ± 0.2	Glucose (mg/dL)	571	431–628
Albumin (g/dL)	4.3 ± 0.4	AST (U/L)	20	15–30
Na (mmol/L)	130.7 ± 4.7	ALT (U/L)	18	13–25
Ca (mg/dL)	9.6 ± 0.6	SIRI	4.4	1.7–8.5
Cl (mmol/L)	103.7 ± 7.1	SII	2424	894–3323
K (mEq/L)	4.7 ± 0.8	Urine Ketone	3	2.0–3.0
HALP	4.6 ± 1.9	Urine Glucose	3	3.0–4.0

**Table 2:** Relationship Between ICU Admission and Blood Parameters, Inflammatory Indices, and HALP Score.

	* *p* Value		** *p* Value
Hb (g/dL)	0.633	Wbc (10^3^/uL)	0.272
Hct (%)	0.316	Mcv (fL)	0.682
Lymphocyte (mcL)	0.826	Neu %	0.736
Plt (10^3^/uL)	0.406	Neutrophil (mcL)	0.046
PO_2_ (mm Hg)	0.385	Monocyte (mcL)	0.018
PCO_2_ (mm Hg)	0.984	pH	0.005
HCO_3_ (mmol/L)	0.012	Lactate (mmol/L)	0.272
Urea (mg/dL)	0.917	FCoHb	0.419
Creatinine (mg/dL)	0.586	Glucose (mg/dL)	0.598
Albumin (g/dL)	0.821	AST (U/L)	0.11
Na (mmol/L)	0.453	ALT (U/L)	0.208
Ca (mg/dL)	0.354	SIRI	0.001
Cl (mmol/L)	0.664	SII	0.001
K (mEq/L)	0.888	Urine Ketone	0.578
HALP	0.06	Urine Glucose	0.203

* *p* = Student’s *t*-test, ** *p* = Mann–Whitney U test.

**Table 3:** Correlation Between ICU Length of Stay and Inflammatory Indices, HALP Score, and Blood Parameters.

	r	*p*
SIRI	0.513	0.001
SII	0.451	0.004
HALP	−0.261	0.108
Lymphocyte	−0.252	0.122
Monocyte	0.469	0.003
Plt	0.158	0.336
Albumin	−0.074	0.655
Wbc	0.224	0.171
pH	−0.463	0.003
HCO_3_	−0.4	0.012
FCoHb	−0.205	0.21
Neutrophil	0.323	0.045

**Table 4:** Sensitivity and Specificity Values of Inflammatory Indices and HALP Score According to ICU Admission Status in DKA Patients.

	AUC	Sensitivity	Specificity	Youden Index	Cut-Off	*p* Value
HALP	0.251	0.28	0.72	−0.363	5.2	0.11
SII	0.82	0.72	0.857	0.577	2272	0.001
SIRI	0.811	0.76	0.857	0.617	3.911	0.001

**Table 5:** Regression Analysis Between Inflammatory Indices and pH and HCO_3_.

Model	Coefficient	R-Squared	*p*-Value
pH vs. SII	−0.000023	0.1196	0.0310
pH vs. SIRI	−0.010986	0.2926	0.0004
HCO_3_ vs. SII	−0.000191	0.159	0.0437
HCO_3_ vs. SIRI	−0.198028	0.1900	0.0055

**Table 6:** Multivariate Logistic Regression Analysis of pH, HCO_3_, SII, and SIRI in Relation to ICU Admission.

Variable	B (Coefficient)	Standard Error	*p* Value	OR (Exp(B))
pH	−0.689	9.41	0.942	0.50
HCO_3_	−0.423	0.385	0.272	0.65
SII	0.00093	0.00059	0.114	1.0009
SIRI	−0.0276	0.191	0.885	0.97

## Data Availability

The data presented in this study are available on request from the corresponding author. Data is not publicly available due to privacy and ethical restrictions.

## Supplementary Materials

The following supporting information can be downloaded at https://www.mdpi.com/article/10.3390/children12111445/s1.
